# F_4_-Neuroprostanes: A Role in Sperm Capacitation

**DOI:** 10.3390/life11070655

**Published:** 2021-07-05

**Authors:** Cinzia Signorini, Elena Moretti, Daria Noto, Simona Mattioli, Cesare Castellini, Nicola Antonio Pascarelli, Thierry Durand, Camille Oger, Jean-Marie Galano, Claudio De Felice, Jetty Chung-Yung Lee, Giulia Collodel

**Affiliations:** 1 Department of Molecular and Developmental Medicine, University of Siena, Policlinico Santa Maria Alle Scotte, Viale Bracci 14, 53100 Siena, Italy; cinzia.signorini@unisi.it (C.S.); daria.noto92@gmail.com (D.N.); n.pascarelli@libero.it (N.A.P.); giulia.collodel@unisi.it (G.C.); 2 Department of Agricultural, Environmental, and Food Science, University of Perugia, Borgo XX Giugno 74, 06123 Perugia, Italy; simona.mattioli@unipg.it (S.M.); cesare.castellini@unipg.it (C.C.); 3 Institut des Biomolécules Max Mousseron (IBMM), UMR 5247, CNRS, Université de Montpellier, ENSCM, 34090 Montpellier, France; thierry.durand@umontpellier.fr (T.D.); camille.oger@umontpellier.fr (C.O.); jean-marie.galano@umontpellier.fr (J.-M.G.); 4 Child Neuropsychiatry Unit, Azienda Ospedaliera Universitaria Senese, 53100 Siena, Italy; geniente@gmail.com; 5 Neonatal Intensive Care Unit, Azienda Ospedaliera Universitaria Senese, 53100 Siena, Italy; 6 School of Biological Sciences, The University of Hong Kong, Hong Kong, China; jettylee@hku.hk

**Keywords:** capacitation, CASA, human sperm motility, F_4_-neuroprostanes, Phospho-AMPKα

## Abstract

F_4_-neuroprostanes (F_4_-NeuroPs), derived from the oxidative metabolization of docosahexaenoic acid (DHA), are considered biomarkers of oxidative stress in neurodegenerative diseases. Neurons and spermatozoa display a high DHA content. NeuroPs might possess biological activities. The aim of this in vitro study was to investigate the biological effects of chemically synthetized 4-F_4t_-NeuroP and 10-F_4t_-NeuroP in human sperm. Total progressive sperm motility (*p* < 0.05) and linearity (*p* = 0.016), evaluated by a computer-assisted sperm analyzer, were significantly increased in samples incubated with 7 ng F_4_-NeuroPs compared to non-supplemented controls. Sperm capacitation was tested in rabbit and swim-up-selected human sperm by chlortetracycline fluorescence assay. A higher percentage of capacitated sperm (*p* < 0.01) was observed in samples incubated in F_4_-NeuroPs than in the controls. However, the percentage of capacitated sperm was not different in F_4_-NeuroPs and calcium ionophore treatments at 2 h incubation. The phosphorylated form of AMPKα was detected by immunofluorescence analysis; after 2 h F_4_-NeuroP incubation, a dotted signal appeared in the entire sperm tail, and in controls, sperm were labeled in the mid-piece. A defined level of seminal F_4_-NeuroPs (7 ng) showed a biological activity in sperm function; its addition in sperm suspensions stimulated capacitation, increasing the number of sperm able to fertilize.

## 1. Introduction

F_4_-neuroprostanes (F_4_-NeuroPs) are prostaglandin-like molecules generated by non-enzymatic oxidation of docosahexaenoic acid (DHA, 22: 6 n-3). DHA may be non-enzymatically oxidized into different classes of neuroprostanes (F_4_-, D_4_-, E_4_-, A_4_-, and J_4_-NeuroPs) [1,2,3], where each class is composed of different isomeric molecules. Oxidation of DHA will generate eight groups of F_4_-NeuroPs (4-, 7-, 10-, 11-, 13-, 14-, 17-, or 20-series) [4,5,6]. Musiek and colleagues [7] reported that the quantification of F_4_-NeuroPs provides a highly selective quantitative window for neuronal oxidative damage in vivo. Mainly, measurement of F_4_-NeuroPs supplies valuable data in exploring the role of oxidative stress in neurodegenerative diseases [8], and F_4_-NeuroPs are considered accurate biomarkers of oxidative damage in Parkinson’s disease [9].

Human spermatozoa and neurons display a high DHA content; it has been reported that sperm motility, morphology, and concentration are positively associated with DHA levels [10]. As previously reported, the presence of seminal DHA and measurement of the n-6/n-3 polyunsaturated fatty acid (PUFA) ratio [11] are important to assess semen quality [12]. In addition, oxidative stress and polyunsaturated fatty acid oxidation have been reported to be relevant to the semen quality [10,11,12,13,14]. Therefore, it is practical to assess the relevance of F_4_-NeuroPs in male infertility as a promising tool to indicate reproductive capacity.

In this regard, F_4_-NeuroPs were measured in seminal plasma to assess their role in male infertility. No significant differences were found in infertile patients (idiopathic infertility and presence of varicocele) compared to fertile men; however, F_4_-NeuroPs correlated positively with sperm necrosis and negatively with normal sperm morphology, suggesting a relevant role of F_4_-NeuroPs as a potential marker of semen quality [13].

Moreover, in a rabbit model, it has been shown that F_4_-NeuroPs in seminal plasma are affected by diet [15]. They were reported to be present in low levels in the rabbits fed with the control diet compared to those fed a high α-linolenic acid (ALA)-enriched diet, or with a fish oil diet (directly suppling n-3 PUFA such as ALA, which is the precursor of eicosapentaenoic acid (EPA), docosapentaenoic acid [DPAn-3], and DHA in human metabolism). Rabbit buck is the smallest and less expensive laboratory animal model in which almost all the reproductive and toxicological endpoints of humans can be measured.

It should also be taken into account that lipid metabolites have a role not only as biomarkers of oxidative stress in humans but also as biological mediators. On this matter, the products of the IsoP or NeuroP pathway were found to have strong biological actions and may therefore participate as physiological mediators of different diseases [16,17]. In particular, n-6 PUFA derivatives resulting from arachidonic acid (ARA) have prothrombotic and pro-aggregatory properties, whereas n-3 PUFA metabolites resulting from EPA and DHA have anti-inflammatory, antiproliferative, and anti-atherosclerotic activities [18,19]. Furthermore, cardiac antiarrhythmic properties have been reported for 4(RS)-4-F_4t_-neuroprostane [20].

Possible effects of F_4_-NeuroPs on sperm are herein investigated. In particular, the aim of this in vitro study was to examine the biological effects of chemically synthetized F_4_-NeuroPs (4-F_4t_-NeuroP and 10-F_4t_-NeuroP) in human sperm. Sperm motility was assessed in the presence of different concentrations of F_4_-NeuroPs. Sperm motion characteristics were acquired by a computer-assisted sperm analyzer (CASA). The immunofluorescence analysis allowed the localization of Phospho-AMPKα (Thr172), a protein that regulates energy balance and metabolism, which are key functional sperm processes for fertilization. Sperm capacitation was recorded using a rabbit sperm model.

## 2. Materials and Methods

### 2.1. Samples

Semen samples were obtained from 15 healthy male donors (aged 29 to 35 years) recruited at the Department of Molecular and Developmental Medicine, University of Siena, Italy. All participants were informed about the study protocol. In addition, we guaranteed their privacy and that the spermatozoa will not be used for genetic studies or fertilization. All volunteers signed an informed consent form for the in vitro study. As it is an in vitro study, ethical approval was not required by the institution.

Ejaculated human spermatozoa were collected in sterile containers after 3–5 days of sexual abstinence. Briefly, after complete liquefaction of the semen samples at 37 °C for 30 min, macroscopic and microscopic examinations were performed [21].

Healthy New Zealand white rabbit bucks of the same age (8 months) and weight (about 4.5  kg) were raised in the experimental farm of the Department of Agriculture, Food and Environmental Science of Perugia (Italy) and used for semen collection. Specific guidelines for the rabbit bucks and the International Guiding Principles for Biomedical Research Involving Animals [22] were followed. The experiment did not require specific authorization by the ethical committee because the animals were not subjected to stressful treatment causing pain and suffering. Semen collection was performed by using a domelike dummy and an artificial vagina maintained at 37 °C internal temperature and used for chlortetracycline (CTC) fluorescence pattern assay.

### 2.2. 4-F_4t_-NeuroP and 10-F_4t_-NeuroP Chemical Synthesis

**Scheme 1:**
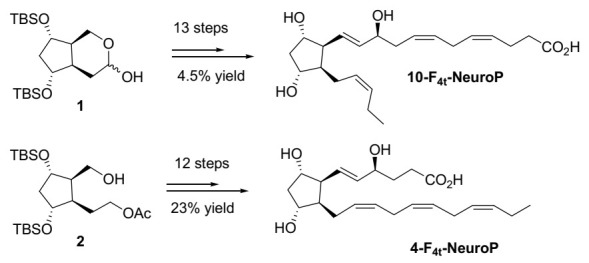
The synthesis of the two series of 4- and 10-F_4t_-NeuroPs.

As 4- and 10-F_4t_-NeuroPs are not commercially available, they were in-house synthesized for the study. [23,24,25] is summarized in Scheme 1.

The two key bicyclic compounds **1** and **2** were obtained in 10 steps and in 8.7% and 10.3% yield, respectively, from commercially available 1,3-cyclooctadiene. The α and ω chains were introduced by using regioselective protections/deprotections and oxidations, followed by a Wittig elongation and cross-metathesis coupling reactions as the main steps for the 10-F_4t_-NeuroP. The free acids were obtained by saponification of the methyl esters in the presence of LiOH. The 4-F_4t_-NeuroP was obtained starting from intermediate **2** in 12 more steps of synthesis leading to a 23% yield, while 10-F_4t_-NeuroP was obtained in 13 steps from intermediate **1** in 4.5% lower yield.

The confirmation of the structures and the purity of these two neuroprostanes were in agreement with the 1H- and 13C NMR, HRMS, and IR experiments, and all these data can be found at doi:10.1002/chem.201002304 and doi:10.1002/chem.201400380.

### 2.3. Neuroprostane Preparation and Determination of Optimal Amount for the In Vitro Study

A 3 ng/mL stock solution of F_4_-NeuroPs (1:1 mixture of 4-F_4t_-NeuroP and 10-F_4t_-NeuroP) was prepared in ethanol and stored at −20 °C. Fresh working solutions were prepared for each experiment. The human semen samples were incubated with different concentrations of F_4_-NeuroP solution (0.7, 7, 70 ng) in Sperm Washing Medium IrvineScientific^®^ (Santa Ana, CA, USA) for 2 h at 37 °C and 5% CO_2_; then, sperm motility was evaluated using a Bürker-Türk hemocytometer (BT, Brand, Wertheim, Germany) counting chamber. Determination of the sperm motility was carried out by simultaneously counting the number of motile spermatozoa (fast progressive, slow, and non-progressive motility) and immotile sperm in 10 fields of a Bürker-Türk counting chamber (volume of one field: 0.004 mm^3^).

Samples without F_4_-NeuroP treatment were used as controls, and samples without F_4_-NeuroP treatment but in the presence of the same percentage of ethanol did not show any difference (data not shown). Specifically, the final percentage of ethanol in the incubation mixture was no more than 0.07%. The experiments were carried out in five basal semen samples.

### 2.4. Swim-Up

The swim-up technique was used to obtain the motile human sperm fraction: 0.5 mL of each semen sample was placed in a sterile conical centrifuge tube and gently layered with 0.5 mL of Sperm Washing Medium. The tubes were inclined at a 45° angle and incubated for 45 min at 37 °C in 5% CO_2_. Then, 0.5 mL of the uppermost medium that contained motile sperm fraction was collected and used for the experiments. Approximately 10 × 10^6^ swim-up-selected sperm/mL were divided into 2 aliquots: one was incubated with F_4_-NeuroPs and another without F_4_-NeuroPs (control). All of the aliquots were kept at 37 °C in 5% CO_2_ for 2 h, and the sperm motility was evaluated as described below.

### 2.5. Computer-Assisted Sperm Analysis

The motion patterns of the human semen samples treated with F_4_-NeuroPs were analyzed using a computer-assisted sperm analyzer (CASA model ISAS, Valencia, Spain). The setup parameters were based on a previous report [26]. For each semen sample, two drops and six microscopic fields were recorded for a minimum of 300 sperm tracks. All semen samples were recorded at 100 Hz frames for 1 s; thus, 12–200 successive images were recorded.

The following sperm motion parameters were evaluated: motility percentage, curvilinear velocity (VCL), straight-line velocity (VSL), average path velocity (VAP), linearity (LIN = VSL/VCL), amplitude of lateral head displacement (ALH), and beat cross frequency (WOB). The experiments were repeated in eight samples.

### 2.6. Sperm Capacitation Patterns in Rabbit Sperm and Swim-Up-Selected Human Sperm

The rabbit semen samples collected in an artificial vagina heated to 37 °C with water were immediately transferred to the laboratory [22] and divided into three aliquots—control, calcium-ionophore-induced, incubation with F_4_-NeuroPs —and evaluated after 2 and 4 h. Swim-up-selected human sperm were incubated with F_4_-NeuroPs and with Sperm Washing Medium alone for 2 and 4 h.

The chlortetracycline (CTC) fluorescence assay was performed as reported by Kaul et al. [27]. The CTC solution was made by dissolving 7.5 M CTC-HCl in a buffer containing 20 mM Tris–H Cl, 130 mM NaCl, and 5 mM cysteine-HCl at pH 7.0. A sperm suspension (100 μL) was put into a 1.5-milliliter foil-wrapped Eppendorf tube, to which 100 μL CTC stock was added. The cells were immediately fixed by adding 8 μL of 12.5% paraformaldehyde to 0.5 mM Tris–HCl buffer (pH 7.5). The slides were prepared by combining 10 μL of the fixed solution with one drop of 1, 4-diazabicyclo [2.2.2] octane dissolved in glycerol/phosphate-buffered saline (PBS) to retard the fading of fluorescence.

A coverslip was placed on top of the slide, and the sperm cells were gently compressed to allow removal of excess fluid. The slides were examined under an epifluorescence microscope (OLYMPUS, USA) using a CH_2_ excitation filter set at 335–425 nm. Three distinct sperm fluorescence patterns were detected: fluorescence over the entire head, which is characteristic of intact cells (non-capacitated); a non-fluorescent band in the post-acrosomal region of the sperm head, which is characteristic of capacitated acrosome-intact cells; and dull or absent fluorescence on the sperm head, which is characteristic of acrosome-reacted cells. Three hundred sperms per sample were analyzed. The experiments were repeated in five sets of human and rabbit samples.

### 2.7. Immunofluorescence Analysis for the Localization of Phospho-AMPKα (Thr172)

Swim-up-selected human sperms, incubated or not with F_4_-NeuroPs, were washed in PBS, smeared on glass slides, air-dried, and fixed in 4% paraformaldehyde for 15 min. After treatment with a blocking solution (PBS–bovine serum albumin (BSA) 1% Normal Goat Serum (NGS) 5%) for 20 min, the slides were incubated overnight at 4 °C with a primary antibody anti-Phospho-AMPKα (Thr172) (Cell Signaling Technology, Danvers, MA, USA) diluted at 1:100; reaction was revealed by an anti-rabbit antibody raised in a goat Alexa Fluor^®^ 488 conjugate (Invitrogen, Thermo Fisher Scientific, Carlsbad, CA, USA), diluted at 1:100. Incubation without the primary antibodies was used as the control. Nuclei were stained with 4,6-diamidino-2-phenylindole (DAPI) solution (Vysis, Downers Grove, IL, USA). Observations were made with a Leica DMI 6000 Fluorescence Microscope (Leica Microsystems, Germany), and images were acquired using the Leica AF6500 Integrated System for Imaging and Analysis (Leica Microsystems, Germany). Two hundred sperms per sample were evaluated. Five samples were analyzed.

### 2.8. Statistical Analysis

Statistical analysis was performed with the statistical software package SPSS Version 23.0 for Windows (SPSS Inc., Chicago, IL, USA). The Kolmogorov–Smirnov test was used to examine if variables were normally distributed. Levene’s test was employed to check the assumption of homoscedasticity. The unpaired two-sample t-test was used to compare the means of two independent groups. For multiple comparison, the differences among the variables examined were checked using a one-way analysis of variance (ANOVA) followed by Bonferroni’s post hoc test. A significance level of *p* ≤ 0.05 was adopted.

## 3. Results

### 3.1. Determination of Human Sperm Motility after F_4_-NeuroP Incubation

Different amounts of F_4_-NeuroPs (0.7, 7, 70 ng) were incubated with human sperm at 37 °C and 5% CO_2_ for 2 h (Table 1). A range from 0.7 to 70 ng of F_4_-NeuroPs was investigated to test the effect of different amounts of F_4_-NeuroPs, whose biological effects on human semen are currently unknown.

In the 7 ng F_4_-NeuroP aliquots, the total and rapid progressive motility were higher than those observed in the non-supplemented control (*p* < 0.05 and *p* < 0.01, respectively); sperm motility was slightly decreased in the presence of 0.7 ng F_4_-NeuroP treatment, while 70 ng F_4_-NeuroP treatment led to significantly decreased total and rapid progressive motility (both *p* < 0.01) compared to those observed in controls. Therefore, in this study, we selected the level of 7 ng F_4_-NeuroPs for subsequent experimentation.

To better evaluate the sperm motility parameter, aliquots of swim-up-selected sperm were analyzed by CASA after 2 h incubation with 7 ng F_4_-NeuroPs. Total progressive sperm motility was significantly increased after incubation with F_4_-NeuroPs compared to the control (50.6 ± 20.4% vs. 39.9 ± 14.5%; *p* < 0.05). Among the sperm motility characteristics, the percentages of curvilinear velocity (VCL, 66.7 ± 13.0% vs. 64.6 ± 12.5%), straight-line velocity (VSL, 43.1 ± 9.6% vs. 38.7 ± 9.6%), straightness (STR, 87.6 ± 7.5% vs. 80.9 ± 4.8%), and wobble (WOB, 74.1 ± 10.7% vs. 70.2 ± 9.0%) were increased after F_4_-NeuroP incubation than those detected in the control, and linearity (LIN) significantly increased (*p* = 0.016) after F_4_-NeuroP incubation compared to the control.

### 3.2. Assessment of Capacitation in Rabbit Sperm, Used as Model, and in Swim-Up-Selected Human Sperm

In order to explain the increase in and type of sperm motility observed in the aforementioned experiments, an induction of capacitation in rabbit sperm was used in the animal model. Aliquots of rabbit semen samples were incubated for 2 or 4 h with calcium ionophore and 2 and 4 h with 7 ng F_4_-NeuroPs. The capacitation rate was evaluated using the CTC fluorescence assay (Table 2).

After 2 h of incubation with calcium ionophore, the percentage of capacitated sperm (71.7 ± 0.9%) was significantly higher than that observed in the control (29.3 ± 2.5%, *p* < 0.001), whereas at 4 h, the percentage of capacitated sperm (58.0 ± 0.3%) was similar to that in the control (53.8 ± 1.9%). The 2-h incubation with F_4_-NeuroPs determined a significant increase in sperm percentage compared to the control (57.8 ± 1.9% vs. 29.3 ± 2.5%; *p* < 0.01). The percentage of capacitated sperm after 2 h of F_4_-NeuroP incubation (57.8 ± 1.9%) was similar to that detected after 4 h in the control (53.8 ± 1.9%).

A significant increase in sperm showing acrosome reaction was detected after 4 h of calcium ionophore incubation (21.3 ± 0.9%, *p* < 0.05) compared to the 4-h control (11.6 ± 1.3%).

Afterwards, an evaluation of capacitation was carried out by CTC analysis in swim-up-selected human sperm. The percentage of capacitated human sperm after incubation with F_4_-NeuroPs (2 h) was significantly higher (42.2 ± 2.5%, *p* < 0.01) than that in the control sample (10.9 ± 1.5%); after 4 h incubation, as found in the rabbit model, a significant increase in acrosome-reacted sperm (34 ± 1.2%) was detected compared to the control (15.2 ± 2%).

### 3.3. Localization of Phospho-AMPKα in Swim-Up-Selected Human Sperm

**Figure 1:**
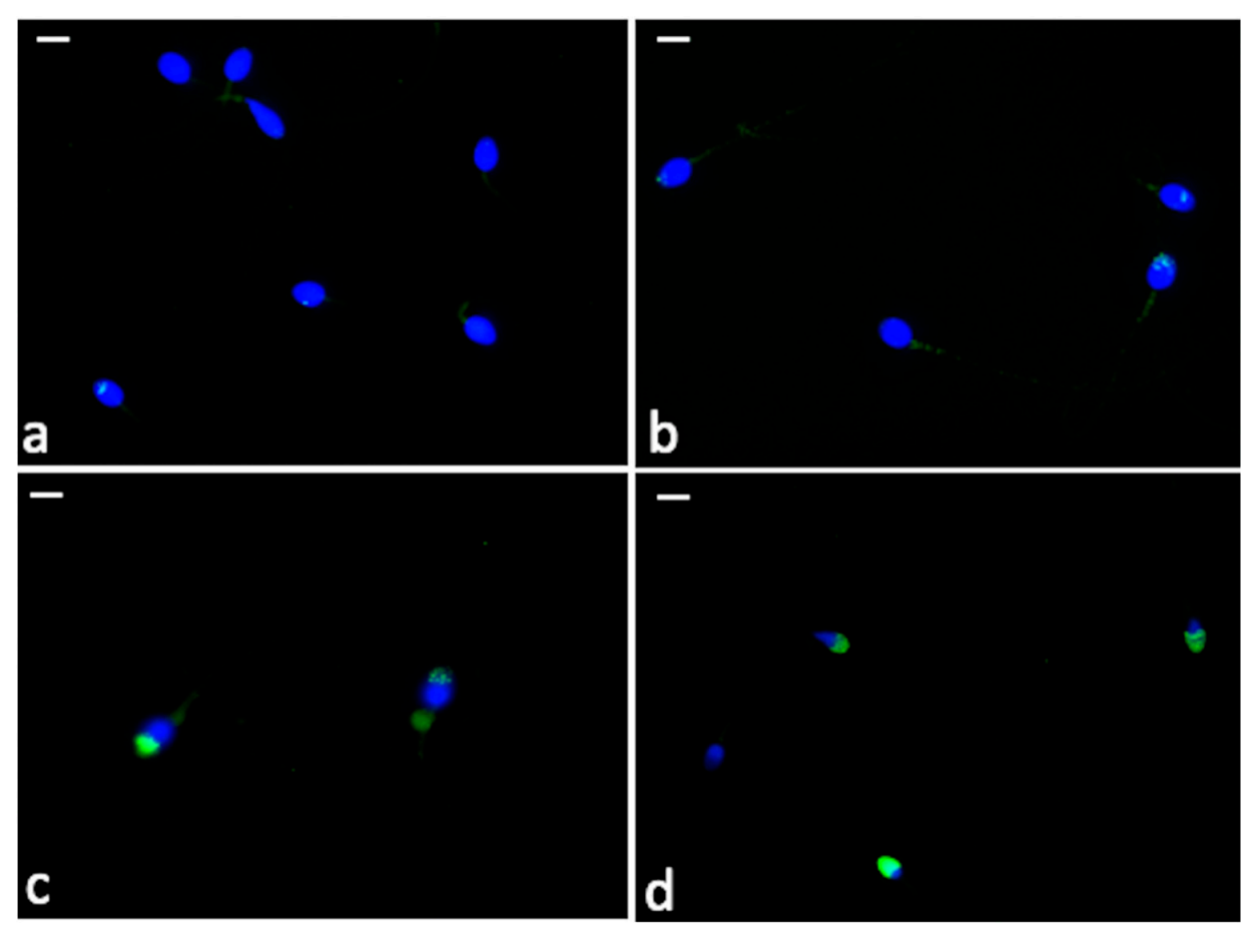
UV micrographs of swim-up-selected sperm incubated with Sperm Washing Medium for 2 h (**a**) or with F_4_-NeuroPs for 2 (**b**) or 4 h (**c**,**d**). In (**a**), the labeling is evident in the mid-piece; in (**b**), it is dotted along the entire tail; in (**c**,**d**), the acrosomes and the mid-piece are fluorescent. Bars: 5 μM.

In control samples, after swim-up selection and 2 h of incubation in Sperm Washing Medium, the phosphorylated form of AMPKα was localized in the mid-piece of a very high percentage of spermatozoa (66 ± 1.8%, Table 3; Figure 1a). This localization was significantly decreased (*p* < 0.01) in samples incubated with F_4_-NeuroPs (2 h, 13.7 ± 1.7%), in which a dotted label along the tail in a significantly increased percentage of sperm (64.7 ± 1%) (Table 3; Figure 1b) compared to the control (0.5 ± 0.5%, *p* < 0.001) was observed. Finally, an intense signal in the acrosomal region of the sperm (57%) was detected after 4 h of F_4_-NeuroP incubation (Figure 1c,d) with respect to the control (17%, *p* < 0.01).

## 4. Discussion

Although NeuroPs have been named “neuro” due to the relative abundance of their PUFA precursor (i.e., DHA) in the brain, NeuroPs have been detected and quantified in plasma [28,29,30], urine [31], and semen [13].

Few data are available about the role of these molecules in seminal fluid. Previously, Longini et al. [13] reported that infertile patients with varicocele had higher levels of seminal F_4_-NeuroPs (154.75 ng/mL) compared to fertile controls (20 ng/mL). In addition, Castellini’s group observed that rabbits supplemented with an n-3 PUFA diet showed improved sperm motion traits and higher levels of F_4_-NeuroPs in their seminal fluid compared to controls [15].

These papers suggest that F_4_-NeuroPs have a biological role in male fertility as well as being markers of oxidative damage, as previously reported in other human diseases [6,28].

It is known that sperm cells are rich in PUFAs, which can undergo auto-oxidation through free-radical-initiated mechanisms in vivo, releasing key products related to pathophysiological events.

NeuroPs, as a class of isoprostanoids originating from non-enzymatic oxygenation of DHA, consist of eight regioisomer series (4-, 7-, 10-, 11-, 13-, 14-, 17-, or 20-series), and thus far, among all the F_4_-NeuroP molecules, 4-F_4t_-NeuroPs and 10-F_4t_-NeuroPs are the most represented [6,28,29].

Our hypothesis was that NeuroPs may also possess biological activities.

Isoprostanes derived from ARA are vasoconstrictors in many species and induce proliferation of endothelial and smooth muscle cells; they mediate their biological effects by activation or inhibition of several prostanoid receptors, including the thromboxane receptor (TP), prostaglandin F_2α_ receptor (FP), prostaglandin E_2_ subtype 3 receptor (EP3), and prostaglandin D_2_ subtype 2 receptor (DP2), and by activation of peroxisome proliferator-activated receptor gamma (PPARγ) [32]. Although less evidence of biological activity is available for F_4_-NeuroPs than for F_2_-IsoPs [6], anti-arrhythmic properties and the ability of heart protection against reperfusion injury have been reported for the non-enzymatic oxidized metabolite of DHA [20,33,34]. More recently, Lee et al. [16] reported a neuroprotective effect of 4-F_4t_-NeuroP through the regulation of pro-resolving lipid mediators and regulation of Nrf/HO-1in SH-SY5Y cells.

The aim of this paper was to study the in vitro effect of F_4_-NeuroPs on sperm function. We tested three F_4_-NeuroP concentrations on human ejaculated sperm and different results were obtained: an amount of 7 ng enhanced the progressive sperm motility, an amount of 0.7 ng did not influence sperm motility, and a quantity of 70 ng reduced it. At present, the biological effects of F_4_-NeuroPs on human semen are currently unknown, and the chosen doses are within the range of F_4_-NeuroP levels measured in human sperm samples. Our data suggest that high levels of F_4_-NeuroPs in semen are toxic and an indicator of oxidative stress taking place, while a moderate level of F_4_-NeuroPs may have a role in physiological activities as an inducer of the capacitation process.

In this study, CASA showed a significant increase in progressive sperm motility and the LIN motion parameter after 2 h incubation with 7 ng F_4_-NeuroPs. It is reported that seminal molecules associated to the capacitation process influence sperm motility and trajectory [35,36]; for example, in porcine epididymal spermatozoa, bicarbonate (HCO_3_^-^) activated a more linear and rapid motility [37].

In vitro studies showed that other molecules such as astaxanthin [38] or fertilization-promoting peptide, angiotensin II, and calcitonin, present in seminal plasma [39], ameliorate sperm functioning by accelerating capacitation and acrosome reactions.

Capacitation [40] includes cholesterol loss from the plasma membrane, a change in intracellular ion concentration, an increase in intracellular pH and hyperpolarization of the plasma membrane, activation of flagellum movement, and changes in the pattern of movement, i.e., hyperactivation, phosphorylation of proteins in tyrosine (Tyr) residues [41,42], and the ability to carry out the acrosome reaction stimulated by a physiological agonist.

Using rabbit as the animal model, the percentage of capacitated sperm after F_4_-NeuroP incubation was compared with that obtained with calcium ionophore and in controls by CTC analysis. The results indicated that the used F_4_-NeuroP concentration in rabbit sperm accelerated the time of capacitation almost similarly to calcium ionophore.

Accordingly, in the experiments carried out with human sperm, the incubation with F_4_-NeuroPs allowed a faster increase in capacitated sperm as compared to the experimental conditions in which F_4_-NeuroPs were not used.

A study on neuroprostanes opened up new perspectives for products of non-enzymatic oxidized DHA as potent mediators in diseases involving ryanodine complex destabilization [20]. Ryanodine receptors (RyR) are present in spermatogenic cells, and ryanodine can modulate the flagellar bend amplitude [43], one of the important characteristics of sperm hyperactivation also linked to Ca^2+^ influx [44,45]. Interestingly, lipid molecules (ceramides) activate RyR and calcium influx through the calcium channel of sperm [46]. Thus, a role for F_4_-NeuroPs in sperm motility might be hypothesized.

Incubation for 4 h in F_4_-NeuroPs led to a significant change in acrosomal status. The acrosome reaction is considered the endpoint of the capacitation process. This is plausible, as we observed a significant difference in reacted sperm with CTC after 4 h. Interestingly, all capacitation events are regulated through the cyclic AMP (cAMP)/protein kinase A (PKA) system [47,48]. Therefore, we performed immunocytochemistry localization of the phosphorylated form of AMPK, which plays an important role in cellular energy homeostasis. In the control samples, after 2 h of incubation, the spermatozoa showed an evident Phospho-AMPKα signal in the mid-piece; at the same time, in the presence of F_4_-NeuroPs, a dotted labeling along the entire sperm tail was detected. Finally, after 4 h of F_4_-NeuroP incubation, a high percentage of sperm highlighted an intense signal in the acrosomal region compared to the control, suggesting that this compound might play a role in increasing AMPK phosphorylation. In human sperm, an increase in the phosphorylation of PKA substrates during capacitation has been described [49,50], while in chicken sperm, AMPK phosphorylation occurs in the flagellum and acrosome [51]. These findings agree with the fact that ATP production is not limited to mitochondrial respiration in the sperm mid-piece but may also have other origins through anaerobic glycolysis [52]. The active phosphorylated form of AMPK results in increased sperm quality, antioxidant ability, and sperm–zona-pellucida binding capacity [53].

## 5. Conclusions

In conclusion, a seminal concentration of 7 ng F_4_-NeuroPs showed a biological activity in sperm function and was able to induce capacitation. These molecules, as suggested for others [39], could be used in a clinical setting. Spermatozoa from infertile men may present a kind of ‘capacitation difficulty’ due to an altered lipid composition of the plasma membrane, which has been described in infertile men [10]. The addition in sperm suspensions would stimulate capacitation, resulting in a high proportion of sperm to enable fertilization. Further studies need to explain the mechanism involved by using germinal cell cultures treated with isoprostanoids to better investigate the potential receptors and putative molecular signaling involved in the F_4_-NeuroP biological activity hypothesized here.

## Figures and Tables

**Table 1:** Means ± standard deviation (SD) of total progressive and rapid sperm motility percentages after incubation with different F_4_-NeuroP amounts (0.7 (F_4_-), 7 (F_4_), 70 (F_4_+) ng) and without F_4_-NeuroPs (control). Five semen samples were analyzed. Statistics are also reported: F_4_-, F_4_, F_4_+ vs. control. *p* < 0.05 is considered significant.

	Control	F_4_-	F_4_	F_4_+	Statistics
Total progressive motility %	29.4 ± 12	27.4 ± 11.3	34.4 ± 12.1	13.6 ± 5.9	Control vs. F_4_ *p* < 0.05 Control vs. F_4+_ *p* < 0.01
Rapid progressive motility %	10.4 ± 4.0	9.8 ± 3.8	16.6 ± 4.2	2.4 ± 1.1	Control vs. F_4_ *p* < 0.01 Control vs. F_4+_ *p* < 0.01

**Table 2:** Means and standard deviations (SD) of capacitated or non-capacitated rabbit sperm percentages under different experimental conditions (incubation for 2 or 4 h with calcium ionophore or incubation for 2 or 4 h with F_4_-neuroprostanes (F_4_-NeuroPs, 7 ng)). Five semen samples were analyzed. Statistics are also reported. *p* < 0.05 is considered significant.

	Samples (n°5)	Time 0	Time 2 h	Time 4 h	Statistics
Non-capacitated sperm %	Control	95.0 ± 1.4	59.4 ± 1.8	34.5 ± 3.2	
	Calcium ionophore	95.6 ± 0.5	22.1 ± 1.3	20.7 ± 0.6	
	F_4_-NeuroPs	95.9 ± 1.2	31.6 ± 2.1	12.6 ± 0.9	
Capacitated sperm %	Control	4.9 ± 1.4	29.3 ± 2.5	53.8 ± 1.9	Control vs. calcium ionophore 2 h Control vs. F_4_-NeuroPs 2 h
	Calcium ionophore	4.4 ± 0.5	71.7 ± 0.9	58.0 ± 0.3	
	F_4_-NeuroPs	4.1 ± 1.2	57.8 ± 1.9	61.7 ± 1.5	
Acrosome-reacted sperm %	Control	0	11.3 ± 4.2	11.6 ± 1.3	Control vs. calcium ionophore 4 h Control vs. F_4_-NeuroPs 4 h
	Calcium ionophore	0	6.3 ± 0.6	21.3 ± 0.9	
	F_4_-NeuroPs	0	10.9 ± 1.2	25.7 ± 0.8	

**Table 3:** Means and standard deviations (SD) of percentages of different Phospho-AMPKα localization in swim-up-selected human sperm. Sperm were incubated for 2 or 4 h with F_4_-neuroprostanes (F_4_-NeuroPs, 7 ng). Five semen samples were analyzed. *p* < 0.05 is considered significant. * *p* < 0.05, ** *p* < 0.01; *** *p* < 0.001.

	Control (2 h)	F_4_-NeuroPs (2 h)	Control (4 h)	F_4_-NeuroPs (4 h)
Mid-piece labeling %	66 ± 1.8 *	13.7 ± 1.7 **	17.5 ± 1.7	3.2 ± 1.2
Dotted tail labeling %	0.5 ± 0.5	64.7 ± 1 ***	51 ± 2.6	15 ± 0.8
Acrosomal labeling %	1.2 ± 0.5	18.7 ± 2.2	17.5 ± 2.1	57.2 ± 1.7 **
Any signal %	33.5 ± 1.3	3.7 ± 1.2	14 ± 1.6	24.5 ± 0.6

## Data Availability

Data are available on request from the authors.
